# User experiences of DiTA (dita.org.au): A database of studies of diagnostic test accuracy

**DOI:** 10.1016/j.bjpt.2025.101568

**Published:** 2025-12-25

**Authors:** Mark A. Kaizik, Aron S. Downie, Mark J. Hancock, Robert D. Herbert

**Affiliations:** a School of Biomedical Sciences, Faculty of Medicine and Health, University of New South Wales, Sydney, Australia; b Faculty of Medicine and Health Sciences, Macquarie University, Sydney, Australia; c Neuroscience Research Australia (NeuRA), Sydney, Australia

**Keywords:** Bibliographic database, Diagnosis, Information system, Physiotherapy, Search engine, User experience

## Abstract

*Background:* The DiTA database indexes primary studies and systematic reviews of the accuracy of diagnostic tests related to physical therapy; however, its usability has not been previously assessed.

*Objective:* (i) To assess the usability (layout, navigation, functionality, content) of DiTA

for typical users working within physical therapy; (ii) to report the volume of user interaction with DiTA.

*Methods:* A Think Aloud usability testing protocol was employed with 25 participants during screenshare teleconference interviews while performing DiTA search tasks. Participants then completed the System Usability Scale (SUS) online. Participants’ comments and interpretations from transcribed interviews were coded into four usability categories. Anonymous data on website user behaviour since DiTA’s inception were collected. The main outcome measures were frequency of comments per category with interpretations made during interviews, and SUS usability scores.

*Results:* Participants most often commented about content (49 % of total), typically with positive sentiment. Participants also frequently commented on DiTA’s functionality typically with negative sentiment. Misinterpretations during search tasks were commonly coded into the functionality category. The SUS score of 70.9 was above the usability benchmark for similar platforms. Participants thought they could learn to use DiTA quickly. Since its launch in 2019, DiTA has averaged 88 visits per day, accessed from almost every country in the world, with most users coming from Brazil.

*Conclusion:* Typical users rated DiTA’s usability as above average, commenting frequently on its content and most often positively. DiTA's functionality was often misinterpreted during search tasks. Nevertheless, participants believed DiTA could be learnt quickly.

## Highlights

• DiTA is a physical therapy-related online diagnostic test accuracy literature database.
• Users in almost every country in the world have accessed DiTA; Brazil ranks 1st.
• User experience was assessed with 25 typical users through search tasks and surveys.
• DiTA could be learnt quickly and scored above usability average (62nd percentile).
• DiTA’s content was its most appealing feature but some functions confused users.

## Introduction

Diagnostic tests are regularly used in physical therapy to increase the certainty of whether a particular pathology is present or absent.^1^^,^^2^ Selecting an appropriate diagnostic test can be difficult for clinicians as there can be several choices available.^3^ Selection can be influenced by factors such as pattern recognition and the use of heuristics,^4^ patient preference for a particular test,^5^ test availability or access,^6^ and fear of litigation and ordering tests as a ‘defensive medicine’ tactic.^7^ Although many factors influence selection, empirical evidence of the test’s accuracy should be an important consideration.^8^ Inaccurate tests may lead to misdiagnosis and inappropriate treatment, including overtreatment and undertreatment, potentially resulting in poor health outcomes and wasted resources.^9^, ^10^, ^11^, ^12^, ^13^

Empirical evidence is provided by primary studies of diagnostic test accuracy and systematic reviews of primary studies of diagnostic test accuracy. Finding this evidence has been difficult for many reasons such as being indexed on multiple databases or requiring different search interfaces or syntax for its retrieval.^14^ Although there are no prevalence data describing physical therapists’ use of diagnostic accuracy evidence to inform clinical decisions, there are known barriers to using evidence-based physical therapy practice that include lack of time (53 % of reported barriers) and lack of access (35 % of reported barriers).^15^ The establishment, in 2019, of the DiTA database (also known as “DiTA”, an acronym for *Di*agnostic *T*est *A*ccuracy) was made in part to mitigate these issues. DiTA was published online with the purpose of indexing primary studies of diagnostic test accuracy and systematic reviews of primary studies of diagnostic test accuracy specifically related to physical therapy. This differs from other biomedical databases such as PubMed^16^ that indexes evidence from multiple health disciplines and not just physical therapy, or to PEDro^17^ that indexes empirical evidence specifically related to physical therapy interventions. When first published, DiTA indexed 979 primary studies and 104 systematic reviews in 16 languages.^8^ Following the initial large-scale search to seed the database, DiTA has been updated monthly^18^ and currently indexes 2222 primary studies and 289 systematic reviews in 19 languages. It is freely accessible for all to use at dita.org.au.

The usability of DiTA has, however, not been assessed, unlike other databases such as UpToDate^19^ (an evidence-based point-of-care medical resource) which has been assessed during physician trainee clinical decision making. In this study, 85 % of respondents described it as easy to use and 88 % agreed they enjoyed using it to look for answers.^20^ The International Organization for Standardization has defined usability as “the extent to which a product can be used by specified users to achieve specified goals with effectiveness, efficiency, and satisfaction in a specified context of use”.^21^ Low usability of DiTA would limit physical therapists’ use of this evidence in their practice as searches would take longer to perform or be less effective.

The primary aims of this study were to describe the DiTA database website usability and report DiTA’s usability rating for the first time. A secondary aim was to report usage patterns of the DiTA website.

## Methods

### Study protocol

The study protocol was prospectively published on Open Science Framework.^22^ All procedures were performed in compliance with relevant laws and institutional guidelines and were approved by the Human Research Ethics Advisory Panel Executive of the [University of New South Wales] on July 20, 2021 (HC210465).

### Study design

The primary aims of the study were investigated using a usability study design^23^ of a representative group of typical users of the DiTA database website. Study participants performed two search tasks then completed a survey of their experiences. The secondary aim of the study was achieved by conducting a retrospective analysis of DiTA users’ search behaviours.

### Participants

When estimating participant numbers needed in a Think Aloud protocol assessing user experience of software users, Nielsen^24^ found up to 85 % of problems were found after five subjects were assessed. In 2019, when looking at web usability data, Nielsen^25^ recommended testing with 20 users when collecting quantitative usability metrics if a 19 % margin of error relative to the mean was desired. Tighter confidence intervals can be obtained studying more users.^26^ For this study, we included 25 participants who met the inclusion criteria and agreed to participate. The inclusion criteria were that the participant was a clinician, researcher, or academic currently working in physical therapy and they could read and understand English at a level allowing them to use DiTA and complete the survey. This allowed for professional diversity amongst the participants. There were no other inclusion criteria applied such as age or level of professional experience, as no previous data existed to describe typical users of DiTA.

Participants were recruited from various sites such as private health clinics, tertiary education institutions, and research facilities. These sites were chosen from the authors’ professional contacts. To meet the study’s secondary aim, anonymous Google Analytics^27^ user behaviour data of the DiTA website from its inception on September 1, 2019 to May 31, 2023 were analysed retrospectively.

### Participant recruitment strategies

An author contacted sites employing potential study participants using a generic email template. A participant invitation letter was attached to the email describing the study and giving contact details of the authors so that those wishing to learn more about the study could make their own contact. Also attached was a Participant Information Statement and Consent Form. Consent was sought in writing using the included consent form. Once a participant contacted the research team, an author explained the study and answered any questions. Once a participant accepted the invitation, an author organised a teleconferencing interview to observe the participant’s use of DiTA and to subsequently conduct a user experience survey (Appendix A). Participants were not reimbursed for participating.

### Procedures

Interviews were conducted by one author (MK) in a location convenient for each participant at a computer with which they were familiar. No training was given to participants on the use of DiTA. For each interview, Zoom teleconferencing software^28^ with screenshare and recording capabilities was used. Participants were asked to nominate two clinical questions related to physical therapy diagnostic test accuracy then attempt to answer each question using DiTA. “Think Aloud” protocols^21^^,^^29^ were employed to assess user experience of DiTA. The author conducting interviews had two hours of training on these protocols from a colleague with previous research experience in their use. Think Aloud protocols involved participants describing aloud their experiences of using the website during the search tasks. The interviewer only engaged with participants to encourage them to continue describing their experience if they remained silent for a period, but did not assist participants through the task. The author took written notes during the search tasks, and made video and audio recordings of the interviews. The videos recorded participants’ screens but not the participants themselves.

Following these two search tasks, each participant was asked to complete the System Usability Scale (SUS) questionnaire (Appendix B).^30^, ^31^, ^32^ The SUS is a 10-item questionnaire with five response options ranging from Strongly Agree to Strongly Disagree and is recognised as a reliable tool to measure website usability.^32^, ^33^, ^34^ The questionnaire yields a single score from 0 to 100 with higher scores representing greater usability.^35^ For this study, SUS questions were modified using simple text substitution to contextualise each question (“this website” was replaced with “DiTA”). The SUS asks users, for example, if they would like to use the website frequently; if they found the website unnecessarily complex; and if they found functions on the website well integrated. Additionally, participants were asked how often they had used DiTA prior to the study.

To review retrospective user behaviour, anonymous Google Analytics data were collected about the DiTA website from inception on September 1, 2019 to May 31, 2023.^36^^,^^37^

### Data extraction and synthesis

Transcripts of the interview Zoom recordings were extracted verbatim using Otter online transcription software^38^ and checked by one author for accuracy. Two authors (MK and AD) independently coded a subset of three interviews to check for consistency, and differences were then discussed and reassessed.^39^ NVivo qualitative data analysis software was used for coding the interviews.^40^ This software allows codes to be tagged to blocks of text in documents then exported for analysis.^41^

Codes for tagging interview transcripts were defined before coding began. The authors used an iterative approach to refine key categories found in user experience literature and applied these categories to data to ensure they were appropriate.^29^^,^^39^^,^^42^^,^^43^ This was a confirmatory process – it was not used to identify new categories. The four pre-defined coding categories were: layout, navigation, content, and functionality (Appendix C). Layout relates to the page display and web elements and how these may affect use of the site (e.g., visibility, form design). Navigation relates to ease of movement between pages or users finding suitable links for content or functions. Content relates to how clear, complete, and necessary users find information on the site including terminology used. Functionality relates to whether certain functions are present or absent and how well functions work on the site.^29^

Each comment was also coded for sentiment: positive, neutral, or negative (Appendix C). An example of positive sentiment relating to layout would be a participant describing how they like search results tabled in reverse date order. An example of neutral sentiment relating to content would be a participant commenting that search results include primary studies and systematic reviews. An example of negative sentiment relating to navigation would be a participant conveying frustration about not being able to find previously selected records.

Interview transcripts were also coded for misinterpretations made by participants as judged by the authors. A misinterpretation was defined as a participant’s action or lack of action that led to an inappropriate or less ideal result.^44^ Misinterpretations were coded using the same four pre-defined comment categories. Examples of misinterpretations included searching fields not fitting the participant’s explicit purpose; using search syntax like wildcards incorrectly; and misinterpreting where a link would lead to.

Inter-rater agreement ranged from 91 % to 100 % for all comment and misinterpretation categories in the subset of three interviews chosen. Most disagreements were not substantive. One example was when both authors selected the same quote but one highlighted a shorter section of text. Another example was where both authors selected the same quote but one tagged the comment as having neutral sentiment and the other tagged it as having negative sentiment. As there was a high level of inter-rater agreement^45^^,^^46^ it was decided just one author (MK) would code the remaining 22 interviews.

Anonymous DiTA website user data were extracted from the Google Analytics platform including information relating to site visit metrics and users. Data from the interviews and the Google Analytics platform were entered into spreadsheet templates. The SUS responses were processed through REDCap,^47^ a secure online survey application. Results from the SUS questionnaires were entered into a purpose-built Excel spreadsheet designed to score the data.^34^^,^^35^ All data were de-identified before extraction.

### Data analysis

For the primary aims, a frequency table of comments with their respective categories and sentiments is presented with exemplars chosen based on frequency of reporting and to assist interpretation of category. Ratios of positive to negative comment sentiments are reported. The frequency of participant misinterpretations and their respective categories are presented. The group’s SUS mean score with 95 % confidence intervals and its range of scores are reported.

For the secondary aims, Google Analytics user data are tabulated. This includes site volume data (e.g. total number of visits since inception); site visit metrics (e.g. average time spent on the site per visit); and geolocation of users.

## Results

Of 25 participants, 22 had used DiTA two or fewer times prior to the study and 13 had never used DiTA before. Fifteen participants were recruited from five different private physical therapy clinics, seven participants from two research centres, and three participants from one university (Table 1).

**Table 1:** Participant characteristics.

Category	Description	Number
Occupation	Clinician	15
	Researcher	10
Previous number of times DiTA used	0	13
	1	6
	2	3
	3	0
	4	1
	≥5	2

### Primary aim: describe DiTA website usability and report its usability rating

Table 2 presents category and sentiment coding results of the 25 interviews. Comments about content were noted most often (161/329; 49 %). They were mostly positive and related to factors such as their search question being answered (“Yeah, that’s probably exactly what I want to look at”), how recently published the studies were (“It’s nice and recent”), the inclusion of systematic reviews (“We’ve got a whole lot of systematic reviews so this is good”), and the availability of abstracts in the search results (“Cool, nice, an abstract”). The ratio of positive to negative sentiment for comments in the content category was 1.6 (64:39). The next largest category of comments related to functionality (90/329; 27 %). However, there were more negative than positive comments in this category – the ratio of positive to negative sentiment was 0.80 (16:20). The categories navigation and layout attracted less comments than for functionality (*n* = 40 and *n* = 38, respectively), with navigation returning a larger ratio of positive to negative comments (3.5; 14:4) than layout (0.76; 13:17).

**Table 2:** Frequency of category and sentiment codes and example comments.

Category and sentiment	Frequency	Representative comment
Layout	38	
Negative sentiment	17	"I’m not sure how this is ordered as well. So probably if I’m doing 15 min search it’s not ideal to try to go over 70 records."
Neutral sentiment	8	"I guess I also look at when the study was done. Just so I have an understanding of how current the research is."
Positive sentiment	13	"I’m going to go probably for the systematic review, it’s good it comes up at the top."
Navigation	40	
Negative sentiment	4	"I can’t find a back button. So I’ll just go search again."
Neutral sentiment	22	"Okay, so I figure I’m searching for something. So I click on the Search button."
Positive sentiment	14	"I like that when I scroll back, it doesn’t erase everything that I just entered."
Content	161	
Negative sentiment	39	"I think what I guess would be helpful would be if it had like a PEDro system where it had a rating with the, you know, out of 10. And so that I know how good the quality of the study is, and how much I can trust it, especially when you’re time poor because sometimes that’s all you have time to look at."
Neutral sentiment	58	"So I click on them and then I’d probably just read their abstracts on the link that’s on the page and be like, 'Oh, yeah, that’s what I want, or, 'No, that’s not quite what I’m looking for.'"
Positive sentiment	64	"We’ve got a whole lot of systematic reviews so this is good."
Functionality	90	
Negative sentiment	20	"And subdisciplines, I guess I would just search musc (musculoskeletal), neuro (neurological). It’d be nice if I could (enter) multiple to a degree."
Neutral sentiment	54	"Maybe I won’t fill in type of reference test."
Positive sentiment	16	"I like how it’s really quick and easy with DiTA to just, like, I don’t need to think about using asterisks or any of those other, like, extensive search when you know, when you look at other sites like Medline and all that, where you have to be really systematic with your search."

Table 3 presents the frequency of misinterpretations per category. Participants were observed making most misinterpretations relating to functionality (45 % of the total) and fewest relating to navigation (8 % of the total). The most common misinterpretations during the search tasks included unnecessarily using multiple search fields when fewer would have been more effective; misinterpreting the two reference test search fields (“Type” and “Name”) as index test fields; misinterpreting dropdown list items; incorrectly spelling search terms; and misinterpreting how to use DiTA-specific search syntax such as wildcards (e.g., *) or Boolean operator functions.

**Table 3:** Frequency of misinterpretation* per category.

Category	Frequency of misinterpretation	Percentage of total
Layout	10	14%
Navigation	6	8%
Content	25	34%
Functionality	33	45%
Total	74	100%

⁎ A misinterpretation is defined as a user’s action or lack of action leading to an inappropriate or less ideal result.

During the interviews there were occasions where the author’s encouragement of the participant to continue describing their experiences may have acted to falsely prompt or guide the search task itself. It was not possible to collect data on these events.

Individual usability scores from the SUS questionnaires (out of 100) ranged from 22.5 to 97.5 with a mean SUS score of 70.9 (95 % CI 64.1, 77.7). When compared to a benchmark website usability mean score of 67,^34^ DiTA’s score of 70.9 was placed in the 62nd percentile, that is, DiTA’s SUS usability score was better than 62 % of websites. The mean SUS score for question 2 (“I found DiTA unnecessarily complex”) in this group was below the usability benchmark. This is supported by comments from participants who had repeated unsuccessful search attempts. Comments ranged from unjustified self-criticism (“Search again. Still no. Could be I’m spelling something wrong. Probably. Okay, well I don’t know what I would do now.”) to frustration (“I’d go to Google!”). However, responses observed showed that many participants quickly learnt how to improve searches during the interview, without assistance, which was also reflected in the group’s SUS score for question 7 (“I would imagine that most people would learn to use DiTA very quickly”) that achieved above the usability benchmark. For example, after unsuccessful searches that were too specific as they used an unnecessary number of fields, participants reflected, “Maybe I’ve got too many of these (filled fields) so I’m just trying to clear them” and “…my strategy here is just to take things off”.

### Secondary aim: report dita website usage patterns

**Fig. 1:**
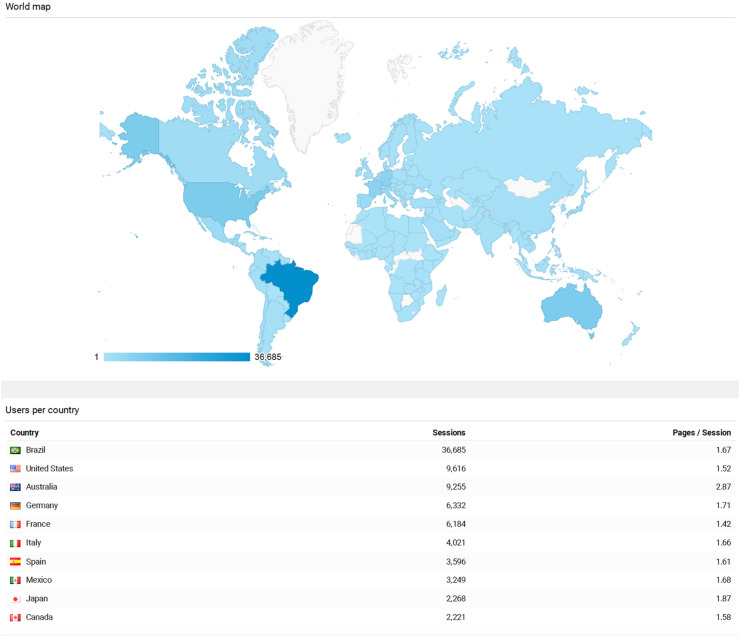
Users of DiTA by country for the period September 1, 2019 to May 31, 2023.

DiTA’s website Google Analytics data from inception on September 1, 2019 to May 31, 2023 are presented in Table 4. There were 120,022 visits during this period, an average of 88 per day spending 1 min 43 s per visit. Fig. 1 shows a world map locating all users during this period and the visit volume for the 10 countries most frequently accessing DiTA. DiTA was used by almost every country in the world, with Brazil the largest user averaging 27 visits per day.

**Table 4:** DiTA’s Google Analytics website traffic data: September 1, 2019 to May 31, 2023.

Category	Metric	Value
Site volume data	Total visits during this period	120 022
	Average visits per day	88
Site visit metrics	Average time on site per visit	1 min 43 s
	Average pages browsed per visit	1.75

## Discussion

This study is the first assessment of usability of the DiTA online database. Participants rated DiTA’s usability above average when compared to similar web platforms, largely commenting on its content and typically with a positive sentiment. However, participants were observed misinterpreting DiTA’s functionality most frequently and were more likely to make negative rather than positive comments related to this category. Nevertheless, we found DiTA could be learnt quickly and had been used in almost every country of the world within four years of its launch.

This study followed a pre-specified publicly accessible protocol.^22^ It used a usability instrument with high reliability^32^, ^33^, ^34^ for assessing website usability on different segments of the physical therapy profession. A Think Aloud protocol allowed concurrent data collection of participant actions and comments; however, a limitation of this method is that interviewer prompting can interfere with users’ thought processes and task performance.^29^ We defined coding categories using an iterative process prior to data analysis. Inter-rater agreement for coding of the first three interviews was rated as high with the remaining interviews coded by a single author.

Another potential limitation of this study is that we intended to report search success frequency but decided against this after observing that search success conflated different elements of the search experience (e.g. questions posed, content of database, site usability) which made interpretation difficult. However, as previously reported, post-test SUS scores and post-task metrics such as completion rates and time taken are only modestly correlated, as has been found with other questionnaires.^34^

Participant inclusion criteria were purposefully made simple to enhance the generalisability of findings. Participants had to work in the field of physical therapy and be able to complete the study tasks in English. This allowed diversity amongst the participants with no limitations set for age, experience, past usage of DiTA, or subspecialty in physical therapy. However, selection bias may have been introduced in this study as all participants were recruited directly or indirectly (e.g. snowballing) from the authors’ professional contacts even though invitations were made at a facility level (not at an individual level) and to a broad range of contacts. Moreover, all participants were recruited from Australia and none from other countries such as Brazil (which had the most users of DiTA in the world for the studied period). This may affect how representative the group of participants was of the typical user of DiTA.

Participants often intended to conduct broad searches however regularly used multiple search fields when using fewer would have been more effective. Moreover, participants repeated the error by adding extra search terms into blank remaining fields for subsequent searches. Participants often did not search using ideal fields, such as searching “Title Only” when “Abstract & Title” was more appropriate. Fields were regularly misinterpreted, particularly the two reference test fields (e.g. filling the “Name of reference test” field with “McMurray’s” when McMurray’s test was the index test, not the reference test). Participants were often unclear about dropdown list values and negatively commented on being limited to one choice (“Can we add more than one here?” and “OK, ‘Subdiscipline’, I’ll go…OK, you can only do one…because I’d quite like to tick musculoskeletal and neurology.”). Mistakes with spelling, Boolean operators and other search syntax also led to frustration and unsuccessful searches. Functionality issues could limit DiTA’s clinical impact by discouraging ongoing, re-visit, long-term, or continued use if users found searching took too long or retrieved inappropriate or insufficient records.

To help solve some of these issues, future research should focus on changing DiTA’s search interface. Artificial intelligence research and deployment companies such as OpenAI^48^ have created large language models (LLM) that could be trained on the closed DiTA database to improve the search experience. A single free-text search field could be used to run DiTA search queries using an LLM to largely circumvent search issues found in our study. Alternatively, other solutions might include offering users auto-suggestions for search terms, marking spelling errors, or providing users with optimal search strategy examples.

Limitations of LLMs include use of outdated datasets, generation of inaccurate content, or lack of transparency of generated content.^49^ Retrieval augmented generation (RAG) for LLMs exposes these models to external knowledge sources to address these limitations and may improve health information retrieval if used with DiTA in this way.^50^ However, using these models with DiTA could still produce incorrect answers and explanations, or biased output related to training data, which may lead to incorrect diagnoses or treatment plans with potentially significant consequences for patient safety.^49^^,^^51^

Participants commented on how helpful it would be to display methodological quality ratings of DiTA’s records, like on PEDro (pedro.org.au),^17^ a widely-used database of robust evidence relating to physical therapy intervention (e.g. “I think what I guess would be helpful would be, it had like a PEDro system where it had a rating with the, you know, out of 10. And so that I know how good the quality of the study is, and how much I can trust it, especially when you're time-poor because sometimes that's all you have time to look at.”). Future research could develop a quality rating scale for diagnostic test accuracy studies. Tools currently exist although they do not reliably quantify study quality^52^ and the most commonly used tools are not intended to calculate quality scores.^53^^,^^54^

## Conclusion

In this first assessment of its usability, the DiTA online database was rated above average by users. Participants mostly commented positively on DiTA’s content, although they were often observed misinterpreting or commenting negatively about functionality. Nevertheless, DiTA could be learnt quickly and had been accessed by users in almost every country of the world within the short time since its launch. Despite an overall positive usability score, DiTA's functional limitations need to be addressed to maximise its accessibility, user retention, and real-world adoption in physical therapy practice.

## Data availability

Data associated with this article are available in the Open Science Framework at https://osf.io/wb7du/?view_only=49777f4f813a48ae9abb976811f29c78.

## Declaration of competing interest

None.

## Figures and Tables

**Appendix A:**
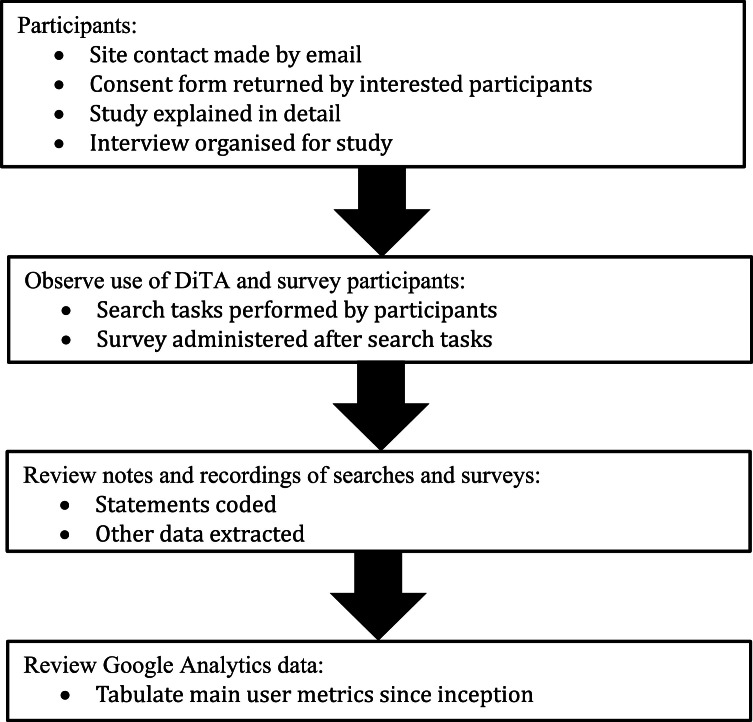
Flow diagram of study design.

**Appendix B:**
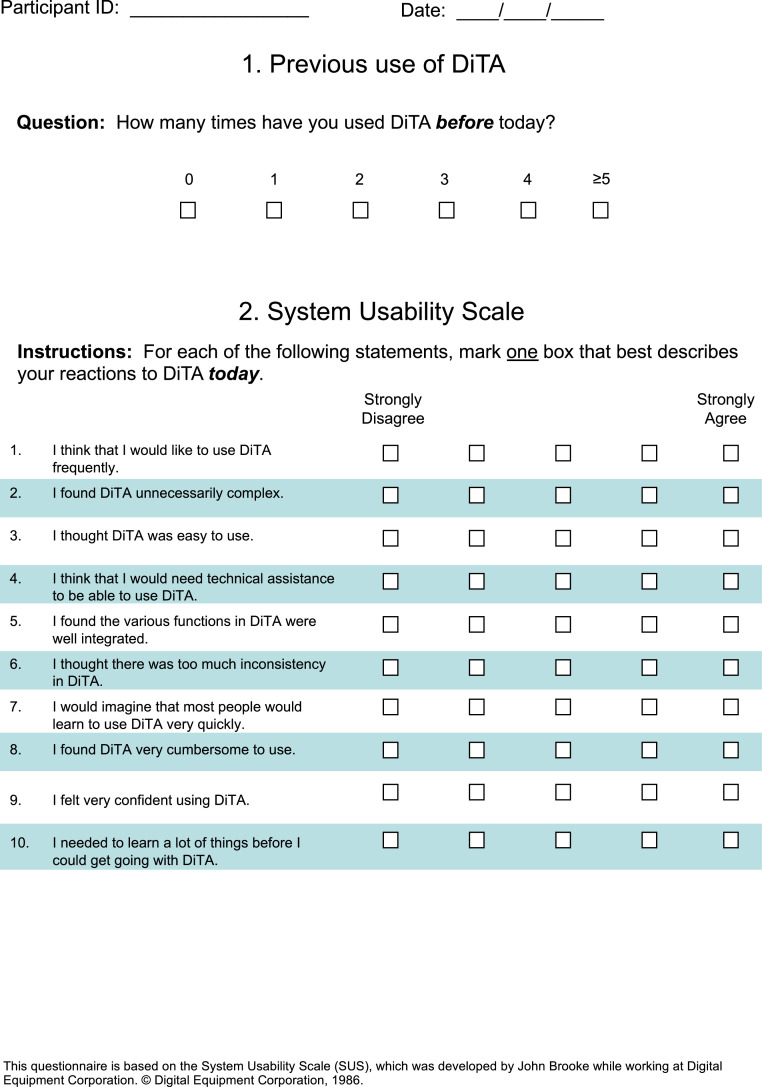
System Usability Scale questionnaire.

## Appendices

**Appendix C:** Definitions and examples of coding categories and sentiments.

*Layout*

Layout relates to the page display and web elements and how these may affect the use of the site (e.g. visibility, form design).

(a) Positive layout example: The participant likes the feature of a dropdown list that is connected to a search field.
(b) Neutral layout example: The participant comments on the search fields being to the left of the screen and the table of results being to the right.
(c) Negative layout example: The participant feels that the font is too small.
(d) Misinterpretation of layout: Any action or lack of action noted by the interviewer relating to the participant's dealings with DiTA’s layout that leads to an inappropriate result (e.g. the participant believes that the search results are presented in order of how robust the studies’ methodologies were).

*Navigation*

Navigation relates to the ease of movement between pages or the finding of suitable links for content or functions by the user.

(a) Positive navigation example: The participant notes that it was really helpful that clicking the "New search" button takes the participant to a new search screen and empties the fields from the previous search.
(b) Neutral navigation example: The participant comments that clicking the "Show saved results" button takes you to a different page.
(c) Negative navigation example: The participant has trouble returning to the home page.
(d) Misinterpretation of navigation: Any action or lack of action noted by the interviewer relating to the participant’s navigation through DiTA that leads to an inappropriate result (e.g. the participant incorrectly believes a link near the search result of a study will take them to a copy of its full text).

*Content*

Content relates to how clear, complete and necessary the user finds the information on the site including the terminology used.

(a) Positive content example: The participant remarks that they are impressed with the number of studies that are found on the site with a search they perform.
(b) Neutral content example: The participant comments on the fact that the search results include both primary studies and systematic reviews.
(c) Negative content example: The participant is disappointed that the site does not include full text versions of the papers that they find in their searches.
(d) Misinterpretation of content: Any action or lack of action noted by the interviewer relating to the participant's dealings with DiTA’s content that leads to an inappropriate result (e.g. the participant doesn’t believe that the database would index research on a particular topic when it actually does).

*Functionality*

Functionality relates to whether certain functions are present or absent as well as how well functions on the site work.

(a) Positive functionality example: The participant likes the fact that a field works with Boolean operators.
(b) Neutral functionality example: The participant comments on how a backspace clears the filled contents of a field that uses a dropdown list.
(c) Negative functionality example: The participant expects an option on ‘Results’ page to be able to sort the results alphabetically by journal name.
(d) Misinterpretation of functionality: Any action or lack of action noted by the interviewer relating to the participant's dealings with DiTA’s functionality that leads to an inappropriate result (e.g. the participant searches for the name of the index test of interest using the “Name of reference test” field).
